# Selenium, Immunity, and Inflammatory Bowel Disease

**DOI:** 10.3390/nu16213620

**Published:** 2024-10-25

**Authors:** James A. Sousa, Derek M. McKay, Maitreyi Raman

**Affiliations:** 1 Gastrointestinal Research Group, Inflammation Research Network, Department of Physiology and Pharmacology, Calvin, Phoebe and Joan Snyder Institute for Chronic Diseases, Cumming School of Medicine, University of Calgary, Calgary, AB T2N 1N4, Canada; james.sousa@bcchr.ca (J.A.S.); dmckay@ucalgary.ca (D.M.M.); 2 Department of Medicine, Cumming School of Medicine, University of Calgary, Calgary, AB T2N 1N4, Canada; 3 Department of Community Health Science, Cumming School of Medicine, University of Calgary, Calgary, AB T2N 1N4, Canada

**Keywords:** selenium, selenoproteins, inflammatory bowel disease, immunity

## Abstract

Dietary intervention is a subject of growing interest in the management of inflammatory bowel disease (IBD), as new incident cases across the globe are rapidly rising, suggesting environmental factors as contributing elements. Dietary components and micronutrients have been associated with IBD pathogenesis or reductions in disease severity. Selenium, a diet-derived essential micronutrient that is important for proper immune system function, has received limited attention in the context of IBD. Selenium deficiency is a common finding in patients with IBD, but few clinical trials have been published to address the consequences of this deficiency. Here, we review the physiological and immunological roles of selenium and its putative role in IBD, and draw attention to knowledge gaps and unresolved issues, with the goal of stimulating more research on selenium in IBD.

## 1. Introduction

Crohn’s disease (CD) and ulcerative colitis (UC) are the two major forms of inflammatory bowel disease (IBD). The changing epidemiology of IBD and its increased global incidence suggest that environmental factors—diet in particular—may induce or modify disease expression [1]. Large prospective cohort studies have linked dietary behaviours with new IBD onset. For instance, a low intake of dietary fiber, fruits, and vegetables, non-adherence to a Mediterranean-style dietary pattern, and a high intake of processed and ultra-processed foods have been associated with the increased incidence of IBD [2,3,4]. Specifically, components of the Western diet pattern, such as a high intake of red meat and high-fat and -sugar foods, have been correlated with increased mucosal inflammation and decreased mucosal barrier function—key mechanisms of inflammation in IBD [5,6]. Alongside dietary components, micronutrients such as selenium (Se) play a crucial role in immune system function. Selenium deficiency has been linked to impaired immune function [7] and is a common finding in patients with IBD [8,9]. Compared to other micronutrients, the role of Se in the context of IBD has received relatively little attention and is poorly understood. There are some data to suggest that Se can influence the immune system and protect against rodent models of colitis, such as dextran-sodium-sulfate-induced colitis. However, clinical data are still developing, and existing data are limited to three trials with limited success, the details of which will be expanded upon in later sections. Therefore, the aim of this review is to explore the physiology of Se absorption and metabolism, as well as its impact on the immune system and IBD pathophysiology.

## 2. Selenium Intake, Absorption, and Metabolism

Selenium is a trace element that is essential for human health. It has unique antioxidant abilities and plays a role in immune system homeostasis, cardiovascular health, and fertility [10]. Selenium is obtained exclusively through the diet, and its intake and bioavailability depend mostly on the molecular form of Se consumed. Brazil nuts, cereals, meat, wheat, fish, and seafood are excellent sources of Se [11] (e.g., the consumption of fish correlates with Se status [12]). Adopting a Mediterranean diet, in contrast to a Western diet, increases Se intake; however, this did not result in a change in serum Se levels in healthy elderly people [13], raising several questions regarding Se metabolism governing homeostasis in both health and disease.

The selenium content in foods can vary widely due to soil concentrations and the bioavailability of Se to plants, which is influenced by a variety of environmental factors (e.g., soil pH and temperature) [14]. The molecular form of Se consumed—organic or inorganic—also influences its bioavailability. Organic Se consists of the selenoamino acids, selenomethionine (SeMet) and selenocysteine (Sec). Foods that contain high concentrations of organic Se include Brazil nuts and wheat [15], whereas inorganic Se includes forms such as selenite and selenate, which are found in foods such as fish and cabbage. Organic forms of Se are absorbed quicker and have a greater bioavailability than inorganic forms [11,15,16]. However, once absorbed into the bloodstream, there is no preference for organic or inorganic Se regarding incorporation into Se-containing proteins (selenoproteins) [17].

**Figure 1:**
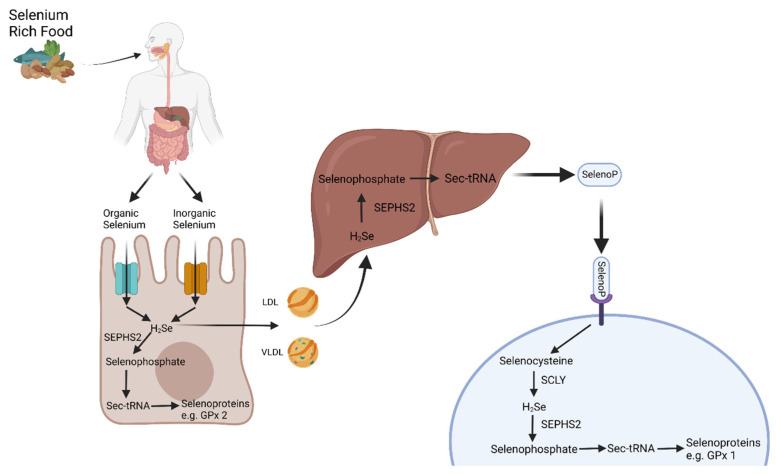
Selenium absorption and metabolism. The consumption of selenium-rich food leads to the absorption of organic and inorganic forms of Se in the gastrointestinal tract through transporters, depending on their molecular form. The absorbed Se is converted into H_2_Se and can be used by enterocytes for the production of selenoproteins or transported to the liver for the production of SelenoP. The transport of Se to other tissues is largely mediated through SelenoP that is then degraded and used in the synthesis of selenoproteins. The catabolism of SelenoP results in the release of selenocysteine that is subsequently converted by SCLY into H_2_Se, which, in turn, is converted by SEPHS2 into selenophosphate, which can then be incorporated into new selenoproteins. H_2_Se, hydrogen selenide; SEPHS2, Selenophosphate Synthetase 2; Sec-tRNA, Selenocysteine transfer RNA; GPx, Glutathione Peroxidase; LDL, low-density lipoprotein; VLDL, very low-density lipoprotein; SelenoP, Selenoprotein P; SCLY, Selenocysteine Lyase. Created in BioRender. Mckay, D. (2024) BioRender.com/o96h565 (accessed on 1 October 2024).

Selenium absorption occurs preferentially in the duodenum, cecum, and colon [11]. SeMet and Sec are taken up into enterocytes by active transport through the amino acid transporters SLC7A9 and SLC3A1, while selenate is absorbed by passive transport through anion exchangers in the SLC26 family [11,18]. The mechanism of selenite uptake is unclear, however, there is some evidence that it is absorbed by passive diffusion through the paracellular space [19]. Once inside the enterocytes, Se can be transformed into hydrogen selenide, bind to low-density lipoprotein and very-low-density lipoprotein particles, and be transported to the liver [11] (Figure 1). In the liver, Se is then incorporated into Sec. The synthesis of Sec occurs co-translationally through the specialized tRNA coded by the UGA stop codon. A Sec insertion sequence encoded in the mRNA is required to prevent the termination of translation. Serine is initially attached to the tRNA and is converted into Sec, which is then incorporated into selenoproteins [20].

The most abundant selenoprotein produced by the liver is Selenoprotein P (SelenoP). Most selenoproteins typically contain only one Sec residue, whereas SelenoP contains ten Sec residues and is the major transport form of Se. A large proportion of plasma Se is in the form of SelenoP, which accounts for up to ~45% of the plasma Se content in healthy humans [21]. SelenoP is transported through the circulation and can bind to receptors such as apolipoprotein E receptor-2 expressed in the testes and brain and megalin in the kidneys [18]. The delivery mechanisms of Se to other tissues are unknown, although not all tissues are treated equally. There is a hierarchy of preferential supply, with the brain and endocrine organs (e.g., the testes and thyroid gland) at the top, followed by the kidneys and other organs such as the liver at the bottom [22]. Once in the organ of interest, SelenoP can be broken down to release Sec for the production of selenoproteins.

### 2.1. Selenoprotein Function

Twenty-five selenoproteins are encoded in the human genome and they affect several cellular processes. A specific function has not been attributed to six of these selenoproteins (Table 1). Generally, selenoproteins are best recognized for their antioxidant capacity. For example, glutathione peroxidases (GPxs) can catalyze a reduction in reactive oxygen species (ROSs) such as hydroperoxides through the oxidation of glutathione, while other selenoproteins play important roles in thyroid hormone metabolism (i.e., iodothyronine deiodinases) and the transport and storage of Se (e.g., SelenoP).

### 2.2. Toxicity and Severe Deficiency

Seminal work in the 1950s defined Se as an essential micronutrient [20], and the current Health Canada guidelines recommend 55 µg Se/day, with 400 µg/day set as the tolerable upper intake level for healthy adults; beyond this, the potential for toxicity emerges. Risk factors for Se toxicity include high concentrations in food supply, accidental or intentional ingestion, or over supplementation [18]. When consumed in excess, acute toxicity can develop, with symptoms including abdominal pain, nausea, vomiting, and headache [11]. Chronic toxicity can occur with a prolonged excessive intake of Se, and this selenosis can manifest as hair and nail loss, dermatitis, and cellular necrosis in the liver and kidney [11]. In the absence of Se measurements, the excess consumption of Se can also lead to breath with a garlic-like odour due to the excretion of volatile Se metabolites [33]. The different molecular forms of Se also differ in terms of their toxicity; hydrogen selenide—used mainly in manufacturing—is classified as highly toxic, whereas the more common selenate and selenite are less toxic [34]. Populations at risk for Se toxicity include those living in areas with highly seleniferous soil and those taking Se supplements, as well as those adjacent to hazardous waste or coal burning plants with Se contamination [35].

At the other end of the spectrum, an estimated 1 billion people have an inadequate intake of Se, and this figure is projected to increase as soil Se contents diminish due to the effects of climate change [36]. The prevalence of Se deficiency varies significantly geographically due to differences in Se content in the food supply, with European countries such as the United Kingdom (29–39 μg/d), Germany (35 μg/d), and France (29–43 μg/d) tending to have a lower Se intake in comparison with Canada (98–224 μg/d) and the USA (106 μg/d) [14]. In some regions of China, the Se intake can be as low as 7 μg/d [14]. Severe Se deficiency has been found in regions where the soil Se content is extremely low. For example, in rural regions of China, as a consequence of low Se intake, there is a higher risk of developing Keshan disease, a cardiomyopathy [37]. Similarly, Kashin–Beck disease, a chronic osteochondropathy, has been observed in northwestern and southeastern regions of China and also been associated with Se deficiency [38]. In addition, individuals who are pregnant, smoke, have genetic disorders (e.g., phenylketonuria), and specific gastrointestinal, muscle, and inflammatory disorders are at an increased risk of Se deficiency [39]. Selenium replacement strategies include balanced dietary approaches and supplementation as needed [18]. Fortunately, through Se supplementation of the food supply, the prevalence of severe deficiency in China is declining [37,38].

### 2.3. Biomarkers of Selenium Status

To assess Se status, multiple approaches should be considered, including oral intake, tissue concentration, selenoprotein function, and excretion (Table 2). The simplest measure is that of Se dietary intake using validated food frequency questionnaires together with 24 h dietary recall questions. However, this method presents challenges in accurate data collection due to the heterogeneity of Se content within similar food sources, as previously described. Further, validated food frequency questionnaires’ capture subjective dietary behaviours and are subject to recall bias. Other factors such as genetics, heavy metal intake, host microbiota, and gut absorptive capacity can all influence Se status, independent of dietary intake [11,40,41]. For example, it has been proposed that Se can form inert conjugates with mercury that can be excreted, reducing Se status and mercury toxicity [41]. Furthermore, under limited Se conditions, the host may compete with their microbiota for Se [42]. Therefore, objective biomarkers of Se status need to be considered. Measures of Se excretion and intake may estimate the retention of Se. Selenium is excreted in the urine and feces [11]. Selenium metabolites in the urine include methylated products such as trimethylselenonium ion (CH_3_Se^+^) and the selenosugar 1β-methylseleno-*N*-acetyl-D-galactosamine, which can be measured to assess excretion [33]. However, these Se metabolites are more representative of recent Se intake rather than Se status [39]. Fecal Se, on the other hand, is composed of undigested or malabsorbed Se. At a very high Se intake, Se can also be excreted through the breath as volatile compounds [33].

Since many organs can retain Se, tissue and blood concentrations can be used to assess Se status. The most widely used body compartment for assessing Se status in humans is plasma [33]. However, total plasma Se may not be a good indicator of Se status due to the non-specific incorporation of SeMet into other proteins such as albumin, which can increase total plasma Se concentrations, but not necessarily influence selenoprotein function or expression [44]. Regarding long-term exposure, nail and hair samples can be used for assessment, however, the use of these tissues is typically limited to a research scenario and is not widely available for clinical practice [11]. Furthermore, hair can be contaminated by Se-containing shampoos.

Lastly, Se status can be estimated through the expression and functionality of selenoproteins. A hierarchy of selenoprotein expression exists, with selenoproteins having housekeeping functions, such as GPx4 and TrxR1 being essential for intracellular redox balance and being expressed during Se deficiency. Other selenoproteins, (e.g., GPx1 and GPx3) are differentially expressed based on Se intake and status. This is due to the preference of different Sec-tRNA isoforms for certain genes. Since the expression of selenoproteins is affected by Se concentration, the activity and expression of specific selenoproteins can be used to estimate Se status. For example, plasma SelenoP concentration can be used to estimate Se status. Other assays exist that can determine the activity of selenoproteins. For example, the activity of GPx3—which is produced by the kidneys and secreted into the plasma—can be measured to inform Se status. These assays offer an advantage over measuring tissue Se concentrations, as they provide information regarding the functional aspect of Se and do not consider non-specific incorporation into other proteins. It is also important to note that GPx activity saturates at a lower Se intake than SelenoP expression, while when measuring serum Se directly, it does not saturate [43]. Therefore, the choice of biomarker used to assess Se status often depends on the expected Se status of the population being assessed [43].

## 3. Selenium and the Immune System

Adequate Se consumption is required for the proper functioning of the innate and adaptive immune systems [45]. For example, selenoproteins such as Selenoprotein K play a role in Ca^2+^ flux in leukocytes, which is important for the oxidative burst and is required for optimal cell activation [46]. Other selenoproteins play a role in redox signaling, specifically in reducing ROS, which has implications for the killing of ingested bacteria and also intracellular signal transduction cascades; evolutionarily, ROSs are likely the original secondary messenger [47].

### 3.1. Leukocyte Migration

Selenium may influence the trafficking of leukocytes to inflamed tissues. Human blood-derived monocytes treated with sodium selenite (2 µg/mL, 16 h) have been shown to decrease the surface expression of L-selectin [48], an important adhesion molecule that binds mucosal vascular addressin cell adhesion molecule 1 on the endothelial cells of gut-associated lymphoid tissue to facilitate migration into the tissue. In addition, Se-treated monocytes display reduced rates of rolling and adhesion under low shear stress and reduce the expression of receptors important for leukocyte extravasation, including intercellular adhesion molecule-1 and vascular cell adhesion molecule-1 [49]. While the evidence is not exhaustive, collectively, it suggests that Se can reduce leukocyte migration out of the vasculature and into tissues. This is a supposition supported by the observation that the Se nanoparticle treatment of mice with dextran sodium sulphate (DSS) colitis resulted in reduced numbers of CD68^+^ macrophages in the colon [50].

### 3.2. Macrophages

There is increasing interest in macrophages as a therapeutic target in IBD [51]. A limited amount of data suggest the Se can affect macrophage polarization, exert an immunosuppressive effect on macrophages, and modify these cells’ contributions to remodeling the extracellular matrix (ECM).

#### 3.2.1. Polarization and Immunosuppression

Selenium can affect macrophage polarization, favouring the development of an anti-inflammatory M2 cell over the pro-inflammatory M1 phenotype. Thus, murine macrophages that were stimulated with 100 nM sodium selenite for 4 days and subsequently stimulated with IL-4 had an increased expression of M2 markers compared to macrophages not exposed to Se [52]. Conversely, cells that were stimulated with LPS to induce the M1 phenotype had a reduced expression of M1 markers when pretreated with Se. Mechanistically, Se incorporation into selenoproteins is important for this effect, as macrophages from glutathione peroxidase 1 (GPx1) knockout (KO) mice have lower arginase activity (a marker of M2 polarization) than wild-type mice when treated with IL-4. This enhanced responsiveness to IL-4 polarization with Se treatment is thought to be due to a variety of effects on eicosanoid class switching and pro-inflammatory transcription factors [53]. These studies are limited to murine models, and future work examining the effect of Se supplementation in human macrophages is needed.

Selenium supplementation has been shown to increase the murine macrophage expression of 15-deoxy-∆^12,14^-prostaglandin J_2_ (15d-PGJ_2_) by modifying the expression of enzymes in the prostaglandin synthesis pathway in murine macrophages [54,55]. Selenium supplementation increases the expression of hematopoietic-PGD_2_ synthase and 15-hydroxy-PG dehydrogenase and decreases the expression of prostaglandin synthases, microsomal prostaglandin E synthase-1, and thromboxane synthase [53]. This results in shifting prostaglandin synthesis away from PGE_2_ and TXA_2_ and towards PGD_2_ and its metabolite 15d-PGJ_2_. Furthermore, 15d-PGJ_2_ has been shown to activate the nuclear receptor peroxisome proliferator-activated receptor gamma, a regulator of the M2 phenotype [52,56,57], and inhibit components of the pro-inflammatory nuclear transcription factor kappa-B (NF-κB) signaling pathway [54]. Lastly, Se treatment can decrease LPS-induced murine macrophage cyclooxygenase 2 and tumour necrosis factor (TNF) expression through the inhibition of mitogen-activated protein kinase signaling pathways [58] and the acetylation of histone H4 [59].

#### 3.2.2. ECM Remodeling

Macrophages’ capability to remodel the ECM can be modified by the expression of selenoproteins. Knockout of the Sec-tRNA in murine macrophages inhibits the production of all selenoproteins, and these cells exhibit a decreased ability to transverse through transwell chambers coated with an ECM-like matrix compared with wild-type macrophages [60]. This did not occur in transwell chambers without the matrix, suggesting that this was not a defect in migration, but rather a decreased capability to degrade and remodel the matrix to allow for passage of the macrophages. The expression of ECM-related genes, including *Collagen type 1 alpha 1*, *Biglycan*, *Metalloproteinase inhibitor 3*, and *Transgelin*, was shown to be significantly increased in Sec-tRNA^−/−^ macrophages. These genes encode for protein components of the ECM and can also inhibit enzymes involved in degrading the ECM, such as matrix metalloproteinases (e.g., A disintegrin and metalloproteinase with thrombospondin motifs 4 and 5). Similarly, *SelenoP* KO macrophages showed decreased migration across EMC-treated transwell filter supports and increased expressions of *Biglycan*, *Metalloproteinase inhibitor 3*, and *Transgelin* [61]. Thus, selenoproteins—potentially SelenoP specifically—may regulate macrophages’ capacity to remodel the ECM. This may be important for intestinal fibrosis in IBD, as Se deficiency has been implicated in fibrosis of the lungs and liver [62,63,64]. Future studies examining markers of intestinal fibrosis in Se-deficient conditions or mice with Sec-tRNA^−/−^ macrophages are needed to further explore this relationship.

### 3.3. Adaptive Immunity

Evidence supporting Se and the adaptive immune response in IBD was demonstrated by a recently published multi-omics study by Huang et al. [65]. Single-cell RNA sequencing found significant enrichment of the pro-inflammatory Type 1 T helper (Th1) cell population in biopsies from patients with CD, and together with metabolomics, demonstrated that Se may contribute to this enrichment. They complemented this with murine studies and suggested that Se can inhibit Th1 cell differentiation in vitro. This was dependent on SelenoW, which was shown to inhibit NF-κB signaling through metabolic changes. The role of SelenoW in suppressing intestinal inflammation is also supported by increased disease activity in SelenoW^−/−^ mice in the DSS colitis model [66]. Huang et al. also assessed the effect of Se supplementation in the adoptive T cell transfer model of colitis, reporting reduced disease activity and a decreased abundance of colon Th1 cells. Lastly, Huang et al. conducted a pilot study where patients with active Crohn’s disease with Se deficiency (Serum Se < 80 μg/L) were administered 360 μg of sodium selenite daily for 8–10 weeks. At the study endpoint, individuals taking the supplement had a reduced clinical disease activity assessed by the Harvey–Bradshaw index, endoscopic scoring (i.e., Simple Endoscopic Score for Crohn’s disease), fecal calprotectin, C-reactive protein concentrations, and abundance of Th1 cells in the colon compared with the control group.

Selenium may influence immune function through several mechanisms, as demonstrated in animal studies. These studies are important for the advancement of our understanding of IBD and allow for assessing factors not feasible with human studies. However, human studies supporting similar findings are lacking, and are necessary in order to develop a full understanding of the impact of Se on the immune system. Differences in the effects of the loss of function of selenoproteins exist between mice and humans, and so the effects of Se on one species may not translate to the other [67]. Considerably more focused research on the putative effects of Se on human immune function is warranted.

## 4. Inflammatory Bowel Disease

### 4.1. Selenium and Rodent Models of Colitis

As proof of concept, rodent models of colitis have been used to investigate the relationships between Se status and IBD. GPx1 and the gastrointestinal-tract-specific GPx2 have been implicated in the prevention of intestinal inflammation. Transgenic mice with a double knockout of GPx1 and GPx2 develop spontaneous colitis [68], characterized by inflammation contained to the mucosa of the ileum and colon only, mucin depletion, inflammatory cell infiltrate, and crypt distortion, whereas mice with a single knockout of either gene were not affected. Unique to this mouse model of colitis, the inflammation was early onset, beginning at around 11 days of age. It is thought that a lack of GPx1 and GPx2 leads to reduced defense against ROS generated mostly by NADPH oxidase 1 and partly by dual oxidase 2, as the knockout of these genes eliminates or reduces pathology, respectively [69,70]. Furthermore, metabolic enteritis can be triggered by polyunsaturated fatty acids (PUFAs) in transgenic mice with a reduced GPx4 expression in their intestinal epithelial cells [71,72]. The enteritis resembles human Crohn’s disease, with small intestinal involvement only and neutrophilic infiltration. GPx4 plays a protective role against lipid peroxidation and subsequent endoplasmic reticulum stress, cytokine release, and inflammation. These data from mouse models are in accordance with a report of decreased GPx4 expression in small intestinal biopsies from individuals with Crohn’s disease [71] and the correlation between PUFA intake and disease activity [72].

The number of studies assessing the effects of Se supplementation on chemical-induced rodent colitis have increased substantially over the past few years and are summarized in Table 3. For example, DSS colitis in mice recapitulates some features of ulcerative colitis, and in one study, disease severity was significantly greater in mice fed with a Se-deficient diet compared to mice given DSS + a Se-sufficient diet [73]. Kaur et al. [74] demonstrated that supplementing the diet with sodium selenite for 8 weeks prior to DSS exposure reduced the severity of murine colitis, as assessed by weight loss, colon length, a disease activity index (DAI), and histological scoring. Similarly, Sang et al. [75] reported that a daily oral gavage of sodium selenite for 30 days prior to DSS treatment reduced colon shortening, weight loss, DAI, and histopathology in treated mice. Contrarily, Hiller et al. [76] reported that pre-treatment with a selenoamino acid or sodium selenite did not affect DSS colitis, and that short-term sodium selenite treatment exacerbated DSS-evoked inflammation.

Organic forms of Se have also been found to protect against DSS colitis and may provide greater benefits than sodium selenite. The use of selenocysteine- or selenocysteine-rich diets was more effective in reducing clinical signs, macroscopic disease scores, and histopathology in DSS colitis compared to mice whose diet contained sodium selenite [77]. Similarly, Se-enriched dietary fiber has been shown to be more effective than sodium selenite in reducing disease activity in the DSS model [83].

Novel forms of Se delivery have also been developed in recent years, including Se nanoparticles (SeNPs) and Se-enriched probiotics. SeNPs have a lower toxicity and are more targeted in their delivery, making them more suitable for supplementation, and they have been shown to be more effective than sodium selenite in protection against DSS colitis [84,89]. Furthermore, a diet supplemented with SeNPs coated with a polysaccharide from the algae *Ulva lactua* or hyaluronic-acid-modified SeNPs has been shown to result in a less severe murine DSS colitis [50]. In terms of Se-enriched probiotics, Se-enriched *Lactobacillus paracasei* has also been shown to be more effective at alleviating DSS colitis than sodium selenite supplementation [80]. The effectiveness of Se-enriched probiotics in treating colitis has also been shown with Se-enriched *Bifidobacterium longum* DD98, *Pichia kudriavzevii,* and *Lactobacillus acidophilus* [90,91,92].

These anti-colitic effects of Se are hypothesized to occur via the inhibition of NF-κB signaling [50,78,84,87], inhibition of cyclooxygenase 2 expression, and suppression of pro-inflammatory prostaglandin synthases [74]. Further, genetically engineered mice whose macrophages selectively lack selenoproteins were not protected from DSS colitis by dietary supplementation with Se, suggesting that macrophages mediate the protection induced by Se supplementation [53]. Many of the studies mentioned above suggest that selenium works through modifying the microbiota, resulting in an increased abundance of beneficial taxa and the production of short-chain fatty acids [79,82,83,84]. Se has been shown to modify the gut microbiota away from dysbiosis, and could affect IBD pathogenesis indirectly [11]. However, the changes seen in the colitis models could be a result of reduced inflammation, rather than Se being the cause of a modified microbiota leading to less severe disease. Studies exploring Se supplementation in gnotobiotic models are needed to help elucidate this relationship. Se supplementation has also been shown to increase antioxidant capability, leading to protection against oxidative stress and subsequent intestinal cell death through apoptosis and ferroptosis pathways [81,84,85], both of which can be elevated in IBD [93,94].

There is a reasonable amount of data regarding Se supplementation in chemical-induced models of colitis, specifically the DSS model. However, these models have their limitations, such as the inability to replicate the chronic and transmural inflammation seen in Crohn’s disease. Therefore, other models, such as those with the spontaneous development of colitis (i.e., IL-10^−/−^ mice) or ileitis (i.e., TNFΔ^ARE^ mice), need to be considered to fully examine the effect of Se in IBD and help to translate these results to humans.

### 4.2. Assessment of Selenium Status in IBD

IBD affects the mucosal integrity of the gastrointestinal tract, including both the small and large intestines; therefore, the absorption of nutrients can be adversely impacted. The most common micronutrient deficiencies identified in patients with IBD include iron, vitamin B12, vitamin D, and Se [9,95]. A study of 83 Korean individuals with UC or CD found that 30% were deficient in Se, as defined by a plasma Se concentration of <95 µg/L [8]. The study concluded that a low serum albumin concentration (<3.3 g/dL) and female sex were associated with Se deficiency. This has been seen in other studies reporting lower serum Se concentrations in females compared to males with IBD, [96] and that plasma Se positively correlates with plasma albumin concentration [96,97]. Lower serum and hair Se concentrations in individuals with CD compared with UC have also been observed in the adult population [96,98,99]. In the pediatric population, a study of 98 and 188 Japanese individuals with CD or UC, respectively, showed that the serum Se concentration was significantly lower in individuals with CD in comparison to those with UC and non-IBD healthy controls [100]. Furthermore, Se deficiency (serum Se < 95 µg/L) was more prevalent in patients with CD than UC (15.3% vs. 5.9%). In a Canadian pediatric population, Se deficiency (definition not defined) was prevalent in 11% and 9.2% of patients with CD and UC, respectively, at diagnosis, and at the 1-year follow up, there was no significant change [101].

The cause of a reduced Se status in patients with IBD is unknown, and results assessing Se intake are limited and mixed. The intake of selenium among patients with IBD in China was lower than in healthy individuals and below the recommended daily allowance (RDA) [102], but in a Canadian CD population, it was well above the RDA (83 µg/d in males and 74 µg/d in females) [103]. In pediatric patients with IBD in New Zealand, there was a reduced intake of Se in comparison to matched sibling controls, but the serum Se was not significantly different between the siblings, which could reflect an altered diet as a response to symptoms [104]. Furthermore, while both Canada and New Zealand have a high prevalence of IBD, the soil Se content and dietary Se intake in Canada are adequate, while in New Zealand, the soil Se content is below the global average [105]. However, due to the globalization of food supply, the local soil Se content may not have a major impact on Se status. Future studies assessing Se intake and status in individuals with IBD are needed to help determine whether the observed Se deficiency is due to a reduced intake due to modified dietary behaviours or is perhaps intrinsic to the disease.

The severity of disease determined by the Harvey–Bradshaw index, the CD activity index, and physician global assessments can be correlated with Se status in CD and UC, where individuals with more severe disease have lower plasma Se concentrations [96,97,106]. Furthermore, the inflammatory biomarker and acute-phase reactant, C-reactive protein (CRP), has been shown to inversely correlate with the plasma Se concentration in patients with IBD, suggesting that Se is an acute-phase reactant [106,107]. An inverse correlation between plasma Se and CRP has also been observed in patients in intensive care units [108], while proinflammatory cytokines have been demonstrated to downregulate intestinal SelenoP biosynthesis by decreasing promoter activity and the expression of selenoprotein synthesis genes (e.g., selenophosphate synthetase 2) [109], further validating serum Se as a potential acute-phase reactant. Objective endoscopic markers of disease activity validate the inverse correlation observed with biomarkers of inflammation in CD [65,106]. Lastly, in a prospective study of 216 individuals with IBD on biologic therapy (70% receiving infliximab (i.e., anti-TNF antibody)), Se deficiency (as measured by serum Se) in individuals with UC (but not those with CD) was associated with future clinical flare-ups of disease [110].

In terms of functional biomarkers of Se status in IBD, reports are inconsistent, with a similar number of studies finding either a decrease or increase in Se status compared to healthy controls (Table 4). Some of the discrepancy between these results likely reflects the variability between the cohorts of individuals assessed (some studies classified whether patients were in remission or had active disease, while most did not) or reflects the degree of inflammation affecting the expression of selenoproteins, and so may not be an accurate indicator of Se status. Alternatively, this discrepancy may be due to geographic variability, as mentioned above in terms of Se intake; however, the relationship between Se intake and status in IBD is not clear.

### 4.3. Genetics

A single-nucleotide polymorphism (SNP) in the selenoprotein gene *GPx1* (rs1050450, C>T) has been identified and associated with IBD. This SNP results in a decreased activity of GPx1 [123]. In a Portuguese population with IBD, the T/T genotype has been associated with an increased risk of UC and increased responses to biologic therapies in patients with CD [124]. On the other hand, in a Polish population, the T/T genotype was associated with a decreased risk of UC and IBD in general [125]. Lastly, SNPs in *SEPSECS*, a gene involved in selenoprotein synthesis, and *SEPHS1* (whose role is not elucidated) have been associated with a lower Se concentration in individuals with CD [126].

### 4.4. Selenium Supplementation and IBD Outcomes

Clinical trials assessing Se supplementation to treat IBD are lacking and, as far as we can determine, are limited to UC. One study examined the effect of eight weeks of ingesting a supplement containing 600 μg of Se along with β-carotene, ascorbic acid, and α-tocopherol in nine individuals with mild-to-moderate UC (definition not provided) [127]. No other treatments were provided. At the study endpoint, four of the nine patients were deemed to be in remission, as determined by stool consistency and sigmoidoscopic scoring. Furthermore, at week four, there was a significant decrease in the mean number of soft or bloody stools per day. However, this study is limited in its sample size, the combination of Se with other antioxidants, the non-use of a validated disease scoring system, and the lack of a control group.

A similar study was conducted with 26 participants with mild-to-moderate UC (based on clinical activity index) taking tablets containing 100 μg of SeMet, 500 mg of curcumin, and 250 mg of green tea extract twice daily for 8 weeks [128]. A significant reduction in clinical disease activity was observed, with 45% of the participants reaching clinical remission at the endpoint. In terms of endoscopic scoring, 69% of the participants saw an improvement (drop of ≥1 on the endoscopic component of the Mayo score) and 25% achieved complete endoscopic remission. Again, the limitations of this study include its small sample size, lack of a control group, and use of a mixture of supplements, which negates any definitive assignment of the beneficial effect solely to Se. For example, curcumin has been shown to be effective in alleviating symptoms of IBD, independent of other treatments [129], and components of green tea are well known to be immunomodulators and anti-oxidants [130]. Yet, we should not dismiss the possibility that Se supplementation for IBD is most effective in combination therapy, a possibility that could be tested.

The largest trial to date was a randomized control trial where 100 patients with mild-to-moderate UC (based on the Simple Clinical Colitis Activity Index) were randomized to take either 200 μg of SeMet or a placebo daily for 10 weeks [131,132]. The participants were excluded if they had received medications such as corticosteroids, non-steroidal anti-inflammatory drugs, anti-TNF agents, or any supplements of Se or other antioxidants within the past 1 month. At the study endpoint, there was no change in the plasma Se concentration in the placebo group, while those taking the Se supplement had significantly increased plasma Se concentrations, going from being deficient to within the normal physiological range. Furthermore, the Se group had a reduced disease activity and increased quality of life, as assessed by the Simple Clinical Colitis Activity Index and the Inflammatory Bowel Disease Questionnaire-9, respectively. Lastly, the Se group had a reduced serum concentration of IL-17, a cytokine that is pro-inflammatory, while serum concentrations of the anti-inflammatory IL-10 were not significantly different between the groups. The authors also proposed that one mechanism of action of Se supplementation is through the upregulation of sirtuin 1 in peripheral blood mononuclear cells. Sirtuin 1 is a negative regulator of NF-κB and has been shown to be downregulated in IBD [133].

Overall, these trials have been limited regarding the form of Se used for supplementation, and there is a gap in how dietary patterns influence Se intake and how this is related to IBD pathophysiology. Currently there are no clinical guidelines regarding assessing Se deficiency and supplementation in IBD from organizations such as the American Gastroenterology Association. Therefore, larger double-blind, placebo-controlled clinical trials are needed to fully determine if Se supplementation is an effective treatment for IBD. These future trials should also include novel supplementation methods such as SeNP and Se-enriched probiotics, which have been shown to be more successful than other forms of Se at reducing disease activity in murine models of colitis. Furthermore, these studies have targeted the induction of remission, so whether Se may play a role in maintaining remission is unknown. Lastly, all these trials have targeted patients with UC, but Se deficiency tends to be more common in patients with CD. Therefore, there is a clear knowledge gap in if/how Se supplementation affects disease activity in patients with CD.

## 5. Conclusions

The physiology of Se status is complex and is influenced by many factors, including dietary behaviour, geography, and pathophysiological processes such as inflammation. Nevertheless, there is mounting evidence to suggest Se as an immunodulatory micronutrient. Selenium deficiency is common in IBD, and published data indicate that Se deficiency can increase the severity of murine colitis, and reciprocally, that treatment with Se can reduce the severity of chemical-induced colitis in mice. However, there are still outstanding questions (Box 1) that need to be answered to inform future research directions and unravel the complex relationships between Se status and intestinal immunity and the effects of Se deficiency and supplementation on IBD activity and disease course. Future research directions include well-designed clinical studies in Se-deficient patients with controlled diets and medical management using Se supplementation, with outcomes of the induction and maintenance of remission. Finally, despite the preliminary nature of many of the data relating to Se in IBD, they are, nevertheless, intriguing, and precisely defining the role of Se in IBD could be a valuable addition to the management options for individuals suffering from UC or CD.

Box 1 **Outstanding questions.**

- What is the optimal biomarker of Se status in patients with IBD?
- Is Se deficiency in patients with IBD due to modified dietary intake or malabsorption?
- How does Se treatment affect the spectrum of innate and adaptive human immune cells?
- How effective is Se supplementation in treating murine spontaneous models of colitis?
- How do we select patients with the best chances of responding to Se supplementation? Does it have a role in the induction or maintenance remission?
- If Se reduces the severity of IBD, what is the cellular mechanism of action and is Se most effective when given as a single supplement or as a component of a combination therapy with other antioxidants or immunomodulators?

## Figures and Tables

**Table 1:** Selenoprotein function and subcellular localization.

Selenoprotein	Subcellular Localization	Role
**Glutathione Peroxidase 1 (GPx1)**	Cytoplasm and mitochondria	Reduction in hydroperoxides, Se storage [23]
**Glutathione Peroxidase 2 (GPx2)**		Antioxidant [24]
**Glutathione Peroxidase 3 (GPx3)**	Secreted	Antioxidant [23]
**Glutathione Peroxidase 4 (GPx4)**	Cytoplasm, nucleus, and mitochondria	Spermiogenesis, reduction in lipid peroxidation [23]
**Glutathione Peroxidase 6 (GPx6)**		Unknown
**Thioredoxin Reductase 1 (TrxR 1)**	Cytoplasm and nucleus	Reduction in thioredoxin [25]
**Thioredoxin Reductase 2 (TrxR 2)**	Mitochondria	Reduction in thioredoxin [25]
**Thioredoxin Reductase 3 (TrxR 3)**		Spermiogenesis [26]
**Iodothyronine Deiodinase 1 (DIO1)**	Plasma membrane	Thyroid hormone metabolism (T4 to T3) [27]
**Iodothyronine Deiodinase 2 (DIO2)**	Endoplasmic reticulum	Thyroid hormone metabolism (T4 to T3) [27]
**Iodothyronine Deiodinase 3 (DIO3)**	Plasma membrane	Thyroid hormone metabolism (T4, rT3, T3 to T2) [27]
**Methionine sulfoxide** **reductase B1 (MsrB1)**	Cytoplasm and nucleus	Antioxidant, actin assembly [28]
**Selenophosphate synthetase 2 (SEPHS2)**		Selenoprotein synthesis [29]
**Selenoprotein F (SelenoF)**	Endoplasmic reticulum	Protein folding quality control [30,31]
**Selenoprotein H (SelenoH)**	Nucleus	Unknown
**Selenoprotein I (SelenoI)**	Endoplasmic reticulum and Golgi	Unknown
**Selenoprotein K (SelenoK)**	Endoplasmic reticulum	ER homeostasis, Ca^2+^ flux in immune cells [31]
**Selenoprotein M (SelenoM)**	Endoplasmic reticulum	Mediate Ca^2+^ homeostasis [31]
**Selenoprotein N (SelenoN)**	Endoplasmic reticulum	Unknown [31]
**Selenoprotein O (SelenoO)**	Mitochondria	Unknown
**Selenoprotein P (SelenoP)**	Secreted and cytoplasm	Transport and storage of Se, antioxidant [32]
**Selenoprotein S (SelenoS)**	Endoplasmic reticulum	Degradation of misfolded [31] proteins
**Selenoprotein T (SelenoT)**	Endoplasmic reticulum	ER homeostasis [31]
**Selenoprotein V (SelenoV)**	Cytoplasm	Unknown
**Selenoprotein W (SelenoW)**	Cytoplasm	Unknown

**Table 2:** Advantages and disadvantages of Se biomarkers.

Marker	Advantages	Disadvantages	References
**Selenium** **Intake**	Simple to assess	Not representative of geographic variability, absorption, or total body status	[33]
**Selenium** **Excretion**	Sensitive for short term exposures	More representative of recent intake	[11,39]
**Tissue and Blood** **Selenium**	Unsaturable Can assess long and short term	Measures non-specific incorporation into non-selenoproteins Requires advanced instruments Subject to contamination	[43]
**Functional** **Biomarkers**	Provides functional information	Saturable Can be influenced by pathophysiological states	[43]

**Table 3:** Selenium supplementation and rodent models of colitis.

Model of Colitis	Form of Se Supplementation	Dose and Mode of Delivery	Length of Time of Supplementation	Results
*Dextran Sodium Sulphate (DSS)*	Sodium selenite	0.5 ppm Se in diet	8 weeks pre DSS treatment	Reduced weight loss, colon shortening, disease activity index (DAI), and histological scoring [74]
	Sodium selenite	2 μg/g body weight Se by oral gavage	21 days pre DSS treatment	Reduced colon shortening, weight loss, DAI, and histological scoring [75]
	Selenocysteine, selenocystine, or sodium selenite	0.9 μg/g body weight Se by oral gavage	During DSS treatment	Reduced weight loss, colon shortening, DAI, and histological damage [77]
	Selenium-enriched phycocyanin	150 μg/g body weight Se-enriched phycocyanin by oral gavage daily	During DSS treatment	Reduced weight loss, bloody diarrhea, colon shortening, DAI, and histological damage [78]
	Selenium nanoparticles (SeNP)	0.8 ppm/d Se by oral gavage	During DSS treatment	Reduced weight loss, colon shortening, DAI, and histological damage [50]
	Selenomethionine or sodium selenite	0.6 μg/g body weight Se in diet	6 weeks pre and during DSS treatment or for 1 week post treatment	No effect other than sodium selenite post-treatment increasing inflammation scoring [76]
	Selenium-enriched yeast	100 μg/g body weight Se yeast by oral gavage daily	During DSS treatment	Reduced DAI, histological scoring, serum inflammatory cytokines. Increased abundance of bacteria in the phylum *Firmicutes*, genus *Bifidobacterium*, and stool butyric acid concentration [79]
	Selenium-enriched *Lactobacillus paracasei*, selenium-enriched yeast, or sodium selenite	0.4 μg Se by oral gavage daily	7 days pre and during DSS treatment	Selenite was less effective than Se-enriched *Lactobacillus paracasei* or yeast [80]
	Total enteral nutrition and Methylselenocysteine (SeMc)	1 μg/g SeMc by oral gavage daily	During DSS treatment	Improved versus DSS and DSS + TEN [81]
	κ-Selenocarrageenan oligosaccharides	1.6, 3.2, or 6.4 μg/g Se by oral gavage daily	7 days pre and during DSS treatment	Reduced DAI, colon shortening, and colon inflammatory cytokines. Increased abundance of bacteria in the genus *Bifidobacterium* and stool butyrate concentration [82]
	Millet-derived selenylated soluble dietary fibre (Se-SDF) or sodium selenite	0.038 μg/g Se by oral gavage daily	28 days pre and during DSS treatment	Se-SDF was more effective than sodium selenite at reducing disease activity [83]
	*Eucommia ulmoides* polysaccharide coated SeNP or sodium selenite	0.5 ppm/d by oral gavage daily	5 days post DSS treatment	SeNP was more effective than sodium selenite and increased antioxidant capacity [84]
	Hyaluronic acid modified SeNP	By oral gavage daily	During DSS treatment starting on day 2	Decreased DAI, colon shortening, tissue inflammatory cytokine, and increased barrier integrity [85]
*2,4,6-trinitrobenzenesulfonic acid (TNBS)*	Sodium selenite	2 μg/g body weight Se in diet	21 days pre-TNBS treatment	Reduced histological damage [86]
	SeNP and silymarin	2 μg/g body weight (SeNP) by oral gavage	During TNBS treatment	Reduced macroscopic and microscopic damage [87]
*Acetic acid*	Sodium selenite and Vitamin E	0.2 μg/g body weight Se by intragastric infusion	During acetic acid treatment	Reduced macroscopic and microscopic damage [88]

**Table 4:** Selenium functional biomarkers in patients with IBD.

Author	Population	Biomarker	Result
Thomas et al., 1994 [111]	Pediatric CD vs. Healthy Control	RBC GPx Activity	Decreased in CD patients
Hoffenberg et al., 1997 [112]	Pediatric IBD vs. Healthy Control	Plasma GPx Activity	Increased in CD patients
Sturniolo et al., 1998 [113]	Adult UC vs. Healthy Control	Plasma GPx Activity	Increased in IBD patients
Tuzun et al., 2002 [114]	Adult IBD vs. Healthy Control	Plasma GPx Activity	Decreased in CD patients
Andoh et al., 2005 [115]	Adult IBD vs. Healthy Control	Serum SelenoP Concentration	Increased in IBD patients
Dincer et al., 2007 [116]	Adult IBD vs. Healthy Control	Plasma GPx Activity	Decreased in CD but not UC patients
Maor et al., 2008 [117]	Adult IBD vs. Healthy Control	Plasma GPx Activity	Increased in IBD patients
Akman et al., 2010 [118]	Adult IBD vs. Healthy Control	Plasma GPx Activity	Decreased in active CD patients
Achitei et al., 2013 [119]	Adult IBD vs. Healthy Control	Plasma GPx Activity	No difference found
Szczeklik et al., 2016 [120]	Adult CD vs. Healthy Control	Plasma GPx Activity	Decreased in remission IBD patients
		Saliva GPx Activity	No difference found
Vaghari-Tabari et al., 2017 [121]	Adult IBD vs. Healthy Control	Serum GPx Activity	No difference found
Barros et al., 2020 [122]	Adult CD vs. Healthy Control	RBC GPx Activity	Increased in IBD patients
